# Renal Cell Carcinoma with Venous Tumor Thrombus: 15 Years of Experience in an Oncology Center

**DOI:** 10.3390/jcm13206260

**Published:** 2024-10-20

**Authors:** Gabriel Faria-Costa, Rui Freitas, Isaac Braga, Maria Ana Alzamora, Sanches Magalhães, João Carvalho, Jorge Correia, Vítor Moreira Silva, Francisco Lobo, Rui Henrique, António Morais

**Affiliations:** 1 Department of Urology, Unidade Local de Saúde de Matosinhos, 4464-513 Matosinhos, Portugal; 2 Department of Surgery and Physiology, Faculty of Medicine of University of Porto, 4200-319 Porto, Portugal; 3 Department of Urology, Instituto Português de Oncologia do Porto, 4200-072 Porto, Portugal; 4 Department of Pathology, Instituto Português de Oncologia do Porto, 4200-072 Porto, Portugal

**Keywords:** renal cell carcinoma, venous tumor thrombus, vena cava, sarcomatoid, clear cell renal cell carcinoma

## Abstract

**Background:** The purpose of this study is to report the experience of a single Portuguese oncology center in the management of patients with renal cell carcinoma (RCC) and venous tumor thrombus (VTT). **Methods:** This is a retrospective analysis of all patients with RCC and VTT surgically treated in our center between 2008 and 2023. Only patients with VTT up to level III (Mayo Clinic classification) were included. Patient, tumor characteristics and peri-operative outcome data were registered. Administration of systemic therapy was performed upon progression. Survival analysis was conducted with the collected data. **Results:** A total of 64 patients (n = 16 women) were included in this study. The mean age at diagnosis was 66.3 ± 10.7 years old. The VTT level was 0, I, II and III in 40 (62.5%), 12 (18.7%), 6 (9.4%) and 6 (9.4%) patients, respectively. Nine patients (14.1%) had distant metastasis at diagnosis. No peri-operative deaths occurred, and the major complication rate was 3.1%. Histology revealed 98.4% of clear cell RCC, with sarcomatoid differentiation present in 12.5% of the cases. A negative margin status was achieved in 54 (84.4%) patients. Systemic therapy was administered in 24 (37.5%) patients during follow-up. The median progression-free (PFS), cancer-specific (CSS) and overall (OS) survival were 23, 60 and 48 months, respectively. In multivariable analysis, significant predictors of CSS were tumor size, sarcomatoid differentiation and collecting system invasion. **Conclusions:** Radical nephrectomy with VTT excision up to level III is a feasible and safe procedure. Patients with large tumor size, sarcomatoid differentiation and collecting system invasion are at the highest risk and should be closely monitored.

## 1. Introduction

Renal cell carcinoma (RCC) presents with venous tumoral thrombus (VTT) in 4–10% of cases [1,2]. In the vast majority of these, the original tumor is a clear cell RCC [1], and it is thought that VTT emerges from micrometastasis that assembles in the vascular system instead of spreading to distant sites [3]. VTT can be limited to the renal vein or rather expand into the inferior vena cava (IVC) and up to the right atrium in 1% of cases [4]. The presence of VTT directly impacts survival, which may vary between 4 and 14 months according to staging at presentation [5]. With surgery, the survival in non-metastatic patients can rise to 40–65% at 5 years for the most favorable tumors [6,7]. Furthermore, vein wall invasion by the tumor is a strong predictor of even worse prognosis and requires surgical excision of the affected vein [6,8]. Other identified prognostic factors are large tumor size, tumor grade, tumor necrosis, sarcomatoid differentiation, perinephric fat invasion, lymph node involvement and distant metastasis [2,9].

The standard treatment for RCC with VTT is radical nephrectomy with thrombectomy [2]. This is a complex procedure that requires extensive pre-operative planning and often a multidisciplinary approach [8]. It is of utmost importance to determine the cephalad extension of VTT by pre-operative imaging. For instance, VTT extending above the diaphragm may require sternotomy for full removal [4]. The most widely used classification for VTT extension was created by the Mayo Clinic and classifies VTT in 0–IV levels [1]. The uniformization of this classification helps to compare results among different centers [3,8,9,10]. There is a considerable mortality associated with the procedure (around 3–16%) and a major complication rate of 6–30% [7,10,11,12]. Higher VTT is associated with higher rates of complications [1].

In this work, we aimed to report our experience in managing patients with RCC and VTT up to level III in an oncology referral center. In addition, we also aimed to identify prognostic factors associated with oncological outcomes.

## 2. Methodology

### 2.1. Study Design and Study Population

The present study is a retrospective analysis of patients with RCC and VTT treated in a Portuguese oncology center (Portuguese Oncology Institute of Porto—IPO Porto) between 2008 and 2023. Data were extracted from the patients’ clinical files. Only patients with histological confirmation of RCC were included. All patients were submitted to radical nephrectomy plus thrombectomy. Patients who did not undergo surgery were excluded from the analysis. This study was approved by the local Ethics Committee (CES—ref. CI-IPOP-52-2024, 16 July 2024).

### 2.2. Peri-Operative Management

Every patient had a contrast-enhanced computed tomography (CT) for staging and to assess the cephalad extension of the thrombus. VTT was categorized according to the Mayo Clinic classification [1]: level 0—thrombus limited to the renal vein; level I—thrombus extending ≤2 cm above the renal vein; level II—thrombus extending >2 cm above the renal vein but below the hepatic veins; level III—thrombus at the level of or above the hepatic veins but below the diaphragm. Patients with level IV classification (thrombus extending above the diaphragm) were excluded from the analysis since these are not treated in our center, owing to the unavailability of cardiothoracic surgery. All patients were previously evaluated by anesthesiology and optimized for surgery. No pre-operative embolization or vena cava filter insertion was conducted. Admission in intensive care was granted whenever necessary. Blood products were readily available. A single dose of low molecular weight heparin was given the day before surgery and restarted 12 h after surgery.

### 2.3. Surgical Technique

For level 0 thrombus, surgery was performed by either an open or laparoscopic transperitoneal approach. All other surgeries were open procedures and were performed by experienced surgeons in our center according to the established surgical principles [8]. Subcostal or chevron incisions were performed at the surgeon’s discretion to obtain the best possible exposure. Early clamping of renal artery was ensured in all procedures. Level 0 thrombi were managed by ligation of the renal vein close to its insertion in the vena cava. For level I thrombi a clamp was applied in the IVC just above the thrombus, and the thrombus was “milked” back and excised alongside with the renal vein. The defect on the IVC was closed with a running 4–0 polypropylene suture. For some level II and all level III thrombi, the assistance of a hepatobiliary surgeon was requested. The liver was mobilized using the Langenbuch maneuver [3], and access to the retrohepatic IVC was gained. In case of a level III thrombus, the Pringle maneuver [3] was also used to avoid liver vascular congestion. Lumbar veins were carefully ligated. The IVC was clamped above and below the thrombus, and the contralateral renal vein was also clamped. The IVC was then opened, and complete removal of the thrombus was performed. If necessary, partial resection and reconstruction of the IVC wall was undertaken. Finally, the cavotomy incision was closed with a running 4–0 polypropylene suture. In cases of well-established collateral circulation, ligation of the IVC was also performed. After removal of the kidney and the thrombus, lymphadenectomy was performed whenever lymph nodes were identified intra-operatively.

### 2.4. Follow-Up

After hospital discharge, patients were followed according to EAU guidelines with CT imaging at the 3rd and 6th month and then every 6 months in the first 3 years and annually thereafter. Lab exams were performed at least twice a year. Disease progression was defined as local recurrence or new metastatic lesions on imaging. Progression-free survival (PFS) was calculated as the time between surgery and either progression or death. Cancer-specific survival (CSS) or overall survival (OS) was calculated as the time between surgery and death attributed to cancer or by any cause, respectively. In case of loss of follow-up, the date of the last evaluation was used.

### 2.5. Study Variables and Statistical Analysis

The following variables were retrieved from the patients’ medical files: patient demographics—age and gender; pre-operative data—symptoms at presentation, smoking status, presence of diabetes, body mass index, Eastern Cooperative Oncology Group (ECOG) performance status score, Charlson comorbidity index, American Society of Anesthesiologists (ASA) score, VTT level and tumor size; operative data—type of surgery (open vs. laparoscopic), segmental resection of IVC, surgical time, estimated blood loss, number of blood units transfused, hemoglobin drop, admission in intensive care and total hospital stay. Histological characteristics of the tumor were also analyzed. The use of systemic therapy in patient follow-up was also registered.

Descriptive statistical analyses were performed using SPSS v. 21.0 (SPSS Inc., 2012). Mean and standard deviation (SD) or median and interquartile ranges (IQRs) were used for continuous variables, while proportions were employed for categorical ones. The t-student and chi-square tests were used to compare continuous and categorical data, respectively. Overall, missing data were 4.8%. There were no missing values for tumor characteristics since we had direct access to pathology reports, and revision of histology was carried whenever necessary. Survival analysis for PFS, CSS and OS were performed by the Kaplan–Meier (KM) method. Univariable and multivariable Cox proportional hazard analyses were conducted to identify independent predictive factors of CSS. The Harrel C-index for the multivariate model was 0.66. A *p*-value below 0.05 was considered for statistical significance.

## 3. Results

### 3.1. Patient Characteristics

A total of 64 patients with RCC and VTT were operated in the 15 years of this analysis. Patient demographics and pre-operative clinical data are summarized in Table 1. There were 16 female and 48 male patients. The mean age was 66.3 ± 10.7 years. Patients were mostly overweighted with a mean BMI of 27.6 ± 5.7 kg/m^2^, and a considerable proportion had diabetes (n = 17, 34.4%) and smoking history (n = 22, 26.5%). Most patients (n = 40, 62.5%) presented with symptoms, most often local symptoms, like hematuria (n = 28, 43.8%) or flank pain (n = 19, 21.9%). Among patients with systemic symptoms, the most common was weight loss (n = 14, 21.8%) followed by asthenia (n = 7, 10.9%). After anesthesiology evaluation, most patients were classified as ASA III (n = 38, 59.4%). The mean Charlson comorbidity index was 5.0 ± 1.6.

### 3.2. Tumor Characteristics

Pre-operative tumor characteristics and post-operative pathological findings are summarized in Table 2. Regarding thrombus extension, the VTT level was 0 in 40 (62.5%) patients, I in 12 (18.7%) patients, II in 6 (9.4%) patients and III in 6 (9.4%) patients. The mean tumor size was 9.8 ± 3.3 cm. Pathology revealed 63 clear cell RCC (98.4%) and one chromophobe RCC (1.6%). Among clear cell RCC, most had nuclear grade 3 (n = 32, 50.8%). Sarcomatoid differentiation and tumor necrosis were present in 8 (12.5%) and 22 (34.4%) of all tumors, respectively. Local staging was pT3a in 41 patients (64.1%), pT3b in 19 patients (29.7%), pT3c (vena cava wall invasion) in 3 (4.7%) patients and pT4 (adrenal gland invasion) in 1 (1.6%) patient. The most common site of local invasion was renal sinus fat (n = 48, 75.0%), followed by perinephric fat (n = 32, 50.0%). Most patients had negative resection margins (n = 54, 84.4%). The most common site of positive margins was the distal end of the renal vein (n = 7, 10.9%). Lymph node removal was performed in 17 patients and showed positive lymph nodes in 3 patients. Upon the time of surgery, 9 patients (14.1%) had distant metastasis identified in the pre-operative imaging. The surgical time, the tumor size and the VTT level were significantly correlated with positive surgical margins (*p* < 0.05).

### 3.3. Surgical and Oncological Outcomes

Surgical outcomes are summarized in Table 3. The mean surgical time was 176 ± 94 min, and the median estimated blood loss was 1.8L (IQR 0.3–2). Segmental IVC resection was needed in 8 (12.5%) patients. The mean hospital stay was 8.9 ± 6.7 days. The tumor size and the VTT level were significantly correlated with surgical time, blood loss and hospital stay (*p* < 0.05). Laparoscopic surgery was performed in 7 (10.9%) patients with VTT level 0. When compared with the open approach in patients with the same VTT level, the laparoscopy approach showed similar baseline features and led to a significant reduction in hospital stay (−4.71 days, *p* = 0.02), estimated blood loss (−758 mL, *p* = 0.001) and intra-operative transfusion (−0.45 units, *p* = 0.029) while maintaining comparable surgical time (+18 min, *p* = 0.287). No patient died within the first 30 days after the procedure. However, one patient died from hemorrhagic stroke at the second post-operative month. Two patients (3.1%) had major complications (Clavien-dindo 4). One patient developed hemorrhagic shock with the need of reintervention for hemostatic revision, and one patient had bilateral pulmonary embolism accompanied by takotsubo cardiomyopathy.

During follow-up, 27 (42.2%) patients had recurrence. Administration of systemic therapy was performed in 18 patients for recurrence and in 6 metastatic (M1) patients. Among different lines of treatment, 25 patients received tyrosine kinase inhibitor (TKI) monotherapy, 6 patients received immunotherapy (IO), 2 patients received a combination of IO/TKI and 8 patients received a mTOR inhibitor. The median PFS was 23 months, and 1y and 5y PFS were 64% and 32%, respectively. A positive margin status was associated with a decrease in the median PFS (−2.95 months, *p* = 0.04).

**Figure 1:**
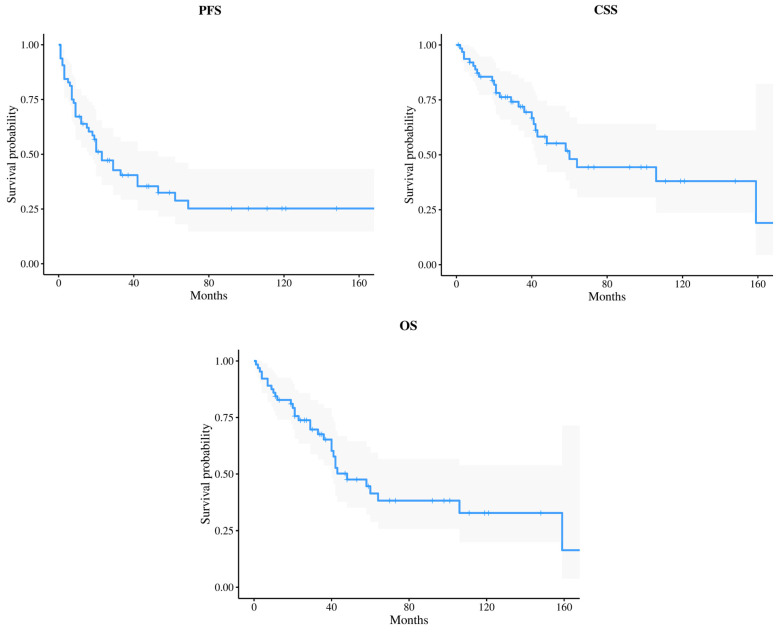
Progression-free survival (PFS), cancer specific survival (CSS) and overall survival (OS) Kaplan–Meier curves (with shadow lines reflecting 95% CI).

Globally, the median CSS and OS were 60 and 48 months, respectively (see Figure 1). The 1y and 5y CSS were 86% and 48%, while 1y and 5y OS were 83% and 42%, respectively. In M0 patients, 1y and 5y CSS were 87% and 53%, while 1y and 5y OS were 85% and 46%, respectively. In M1 patients, 1y and 5y CSS were 75% and 25%, while 1y and 5y OS were 67% and 22%, respectively.

Multivariable analysis for prognostic factors of CSS is displayed in Table 4. In univariable analysis, significant prognostic factors were age, VTT level, tumor size, sarcomatoid differentiation, invasion of collecting system, invasion of adrenal gland and M1 at diagnosis (*p* < 0.05). In multivariable analysis, independent predictors of CSS were tumor size (HR 1.18, 95% CI 1.02–1.35), sarcomatoid differentiation (HR 12.65, 95% CI 3.75–42.66) and collecting system invasion (HR 2.93, 95% CI 1.17–7.33).

## 4. Discussion

In this study, we evaluated the results of 15 years of experience managing patients with RCC and VTT up to level III in a single center. No patient died during surgery, and the major complication rate was 3.1%. Negative margins were achieved in 84.4% of the patients. The median PFS, CSS and OS were 23, 60 and 48 months, respectively. Tumor size, sarcomatoid differentiation and collecting system invasion were independent predictors of worse outcome.

Performing surgery in patients with RCC and VTT has always been challenging for urologists. The first report of surgical management of tumor thrombus reaching the vena cava is attributed to Berg et al. [13] in 1913. Subsequent publications sedimented important anatomical and surgical concepts for planning such complex procedures [14,15]. The survival benefit of an aggressive approach was demonstrated, and it soon became evident that VTT extension was a major determinant for the surgical technique adopted [16]. In 2004, the Mayo Clinic published one of the largest single center series so far, comprising 540 patients, where VTT was classified in levels 0–IV [1]. For each VTT level, they provided detailed surgical techniques that were employed. They reported a mortality rate of 3.2% and a major complication rate of 13%. A higher VTT level was associated with more complications and longer hospital stay. Interestingly, IVC involvement (level > 0) was associated with a lower CSS, but no differences were found among different levels of IVC involvement. The 5-year CSS was 59% for N0M0 patients and 5.8% for N1/N2 M1 patients. Similar results were later reported by Haferkamp A, et al. [17] and Klatte T, et al. [18]. In their series of 111 patients, Haferkamp A, et al. reported a 30-day post-operative mortality rate of 10% and 5-year CSS rates of 45.8% and 6.5% in M0 and M1 patients, respectively. Likewise, Klatte T, et al. analyzed 321 patients and reported a mortality rate of 5%, a major complication rate of 23% and a 5-year CSS of 65% and 19% in M0 and M1 patients, respectively. However, conflicting results were found regarding the prognostic value of the level of IVC involvement. In this regard, a multicentric study with 1192 patients [19] and a population-based analysis of a USA cancer register with 1875 patients [20] do not sustain a prognostic role for the IVC level of involvement, which is in agreement with the Mayo Clinic findings [1]. Likewise, we did not find the VTT level to be an independent predictor of CSS in our series. On the other hand, both patient and tumor characteristics have been pointed out as prognostic factors by the abovementioned studies. A well-structured meta-analysis [2] highlighted the following factors as predictors of CSS: presence of IVC thrombus (vs. level 0 thrombus), large tumor size, high nuclear grade, tumor necrosis, sarcomatoid differentiation, perinephric fat invasion, adrenal gland invasion, positive lymph nodes and metastasis at the time of surgery. Our multivariable analysis results are also in line with these findings, although statistical significance was only reached for some of these factors.

In our series, we found similar outcomes to those reported in the literature. We had no operative deaths and a very low major complication rate. It should be noted that we did not have grade IV tumors in our series, which may have contributed to the positive surgical outcomes. Regarding oncological outcomes, we observed a 5y CSS of 53% for M0 patients and 25% for M1 patients. These figures are in accordance with other series, as already discussed. More recently, Wang T, et al. [21] reported on 350 patients with RCC and VTT. For patients with clear cell histology, they found a 5y PFS and OS of 38% and 56%, respectively. In our series 5y PFS and OS were 32% and 42%, respectively. The larger percentage of sarcomatoid differentiation (12.5% in our series vs. 3.50% in the Chinese series) and the larger tumor size (9.8 cm in our series vs. 7.83 cm in the Chinese series) may possibly explain this slight difference. In fact, sarcomatoid differentiation and tumor size were the strongest predictors of CSS in our multivariable analysis. These factors have been widely reported and are well-established risk factors [2]. Furthermore, our positive margin rate was 15.6%, with most cases due to the involvement of the distal end of the renal vein by the tumor thrombus. Positive margins were associated with an increased risk of progression but were not an independent predictor of CSS. The exact same findings have also been reported by Abel et al. [22]. Nevertheless, these patients should have a close follow-up as timely management of progression may impact their long-term outcome. In our series, no patient underwent adjuvant systemic therapy, but rather upon progression. Overall, 68% patients received TKI monotherapy as first-line treatment, and this was still the most used therapy in subsequent lines. We speculate that oncological outcomes might be different in the era of IO/TKI and IO/IO combination therapy. Yet, there is still no demonstrated survival benefit of adjuvant or neoadjuvant systemic therapy [23,24]. Further studies are eagerly awaited to clarify the role of new generation systemic therapy in RCC patients with VTT.

There is an increasing shift for minimally invasive surgery in the management of RCC with VTT. Currently, available data derive from retrospective studies with few patients. Nevertheless, two meta-analyses have found shorter hospital stay, less blood loss and fewer postoperative major complications with minimally invasive surgery when compared with the standard open approach [25,26]. Oncological outcomes were similar between the two approaches. In our series, 7 patients were operated by a laparoscopic transperitoneal approach. All patients had level 0 thrombus. When compared with the other level 0 patients, the laparoscopic approach showed a significant reduction in hospital stay, estimated blood loss and intra-operative transfusion while maintaining operative times. We expect to have future data to report on the minimally invasive approach in higher-level VTT.

This is a retrospective study with its inherent limitations. The total number of patients was modest and lacked representation of higher levels of VTT. This could have contributed to the modest Harrell C-index of our multivariate model. Nevertheless, our results are in line with other series, which accounts for the generalization of our findings. Furthermore, there might have been technical changes between the years of the analysis and differences between surgeons that we were unable to analyze. However, it is important to mention that this is a single-center study with similar techniques used by all surgeons. Finally, our minimally invasive experience is still limited and is not sufficient to draw major conclusions in this subtopic.

## 5. Conclusions

Management of RCC with VTT up to level III is feasible in an experienced center, with outcomes currently equivalent to those previously reported in other series. Patients with larger tumor size, sarcomatoid differentiation and collecting system invasion warrant a close follow-up. Further studies are awaited to clarify the role of minimally invasive surgery and new generation systemic therapy in the management of these patients.

## Figures and Tables

**Table 1:** Patient characteristics, n = 64.

Variables	Values	Missing Data (%)
Age (years), mean ± SD	66.3 ± 10.7	0
Sex women, n (%)	16 (25)	0
BMI (kg/m^2^), mean ± SD	27.6 ± 5.7	13.4
Smoking history, n (%)	22 (34.4)	10.6
Diabetes, n (%)	17 (26.5)	14.2
Symptoms at diagnosis, n (%)		12.5
*No symptoms*	24 (37.5)	
*Local*	28 (43.8)	
*Hematuria*	19 (29.7)	
*Flank Pain*	14 (21.9)	
*Varicocele*	2 (3.1)	
*Systemic*	14 (21.8)	
*Weight loss*	12 (18.8)	
*Asthenia*	7 (10.9)	
*Hypertension*	2 (3.1)	
ASA score, (%)		6.5
*ASA II*	26 (40.6)	
*ASA III*	38 (59.4)	
Charlson comorbidity index, mean ± SD	5.0 ± 1.6	7.2

**Table 2:** Pre-operative tumor characteristics and pathological findings.

Variables	Values
VTT level, n (%)	
*Level 0*	40 (62.5)
*Level I*	12 (18.7)
*Level II*	6 (9.4)
*Level III*	6 (9.4)
Right side tumor, n (%)	30 (46.9)
Tumor size (cm), mean ± SD	9.8 ± 3.3
Pathology subtype, n (%)	
*Clear cell*	63 (98.4)
*Chromophobe*	1 (1.6)
Nuclear grading, n (%)	
*2*	11 (17.5)
*3*	32 (50.8)
*4*	20 (31.7)
Sarcomatoid differentiation, n (%)	8 (12.5)
Tumor necrosis, n (%)	22 (34.4)
Local staging, n (%)	
*pT3a*	41 (64.1)
*pT3b*	19 (29.7)
*pT3c*	3 (4.7)
*pT4*	1 (1.6)
Local invasion, n (%)	
*Sinus fat*	48 (75.0)
*Perinephric fat*	32 (50.0)
*Collecting system*	23 (35.9)
*Adrenal*	1 (1.6)
Margin status, n (%)	
*Negative*	54 (84.4)
*Renal vein*	7 (10.9)
*Perinephric fat*	2 (3.2)
*Renal Parenchyma*	1 (1.6)
N1 at the time of surgery, n (%)	3 (4.7)
M1 at the time of surgery, n (%)	9 (14.1)

**Table 3:** Surgical outcomes and follow-up.

Variables	Values	Missing Values (%)
Surgical approach, n (%)		0
*Open*	57 (89.1)	
*Laparoscopic*	7 (10.9)	
Segmental IVC resection, n (%)	8 (12.5)	4.7
Surgical time (min), mean ± SD	176 ± 94	4.7
Median estimated blood loss, L (IQR)	1.8 (0.3–2)	9.8
Blood transfusion (units), mean ± SD	1.8 ± 3.3	15.7
Hospital stay (days), mean ± SD	8.9 ± 6.7	0
Intensive care admission, n (%)	15 (23.4)	0
Major complication rate, n (%)	2 (3.1)	0

**Table 4:** Univariate and multivariate analysis of prognostic factors for cancer-specific survival.

Factor	Univariate		Multivariate	
	HR (95% CI)	*p* Value	HR (95% CI)	*p* Value
Age	0.99 (0.95–1.03)	0.482		
Sex (men vs. women)	0.41 (0.19–0.92)	0.031 *	0.67 (0.26–1.69)	0.390
Symptoms at presentation (yes vs. no)	1.84 (0.72–4.69)	0.202		
Side (right vs. left)	0.64 (0.29–1.41)	0.268		
ECOG (3–4 vs. 1–2)	4.41 (0.96–20.32)	0.067		
ASA (3–4 vs. 1–2)	1.58 (0.68–3.68)	0.290		
BMI (≥30 vs. <30 Kg/m^2^)	0.65 (0.25–2.35)	0.771		
Smoking history (yes vs. no)	1.04 (0.43–2.53)	0.993		
Diabetes (yes vs. no)	1.13 (0.45–2.85)	0.800		
Surgical time	1.00 (0.99–1.01)	0.403		
Estimated blood loss	1.00 (1.00–1.00)	0.779		
VTT level (II–III vs. 0–I)	2.45 (1.02–5.88)	0.046 *	1.64 (0.54–4.96)	0.384
Tumor size	1.20 (1.07–1.34)	0.001 *	1.18 (1.02–1.35)	0.022 *
Nuclear grade (3–4 vs. 1–2)	1.57 (0.53–4.61)	0.415		
Tumor necrosis (yes vs. no)	1.07 (0.42–2.71)	0.892		
Sarcomatoid differentiation (yes vs. no)	6.84 (2.46–19.04)	<0.001 *	12.65 (3.75–42.66)	<0.001 *
Perinephric fat invasion (yes vs. no)	1.68 (0.76–3.73)	0.199		
Collecting system invasion (yes vs. no)	4.49 (1.91–10.59)	0.001 *	2.93 (1.17–7.33)	0.022 *
Adrenal gland invasion (yes vs. no)	11.89 (1.39–101.78)	0.024 *	1.6 (0.102–24.98)	0.738
N1 at time of surgery	1.23 (0.66–2.27)	0.519		
M1 at time of surgery	2.53 (1.05–6.08)	0.038 *	2.49 (0.79–7.88)	0.122
Margin status (positive vs. negative)	2.12 (0.84–5.35)	0.113		

ASA = American Society of Anesthesiologists; BMI = body mass index; CI = confidence interval; ECOG = Eastern Cooperative Oncology Group; HR = hazard ratio; VTT = venous tumor thrombus * Statistically significant.

## Data Availability

Data is available upon request to corresponding author.
